# Recurrent Oral Eosinophilic Ulcers of the Oral Mucosa. A Case Report

**DOI:** 10.2174/1874210601812010019

**Published:** 2018-01-29

**Authors:** Norberto Sugaya, Fernanda Martignago, Decio Pinto, Dante Migliari

**Affiliations:** Department of Stomatology, Oral Medicine Division, School of Dentistry, University of São Paulo, São Paulo, SP. Brazil

**Keywords:** Oral ulcerations, Diagnosis, Eosinophilic ulcer, Recurrences, Crohn’s disease, Oral mucosa

## Abstract

**Objective:**

This article describes a case of an Oral Eosinophilic Ulcer (OEU) in an otherwise healthy 31-year-old white woman.

**Introduction:**

The importance of reporting this case was the presence of recurrent episodes with lesions appearing in different areas of the oral mucosa, a type of manifestation not commonly associated with this disease. A typical manifestation of OEU occurs as a single ulceration that goes into healing after an incisional biopsy, a procedure usually required for a proper diagnosis of the disease. In spite of trauma being suggested as the main culprit of OEU, the exact pathogenesis mechanism of this disease remains controversial.

**Case report:**

The pattern of the present case contradicts the usually common course of the disease, as the patient had experienced many recurrent episodes for almost 2.5 years, with the recurrences occurring even after biopsies performed during some of the relapses. Differential diagnosis included recurrent aphthous stomatitis, recurrent intra-oral herpes, autoimmune disease, Crohn’s disease and malignancy.

**Conclusion:**

Fortunately, the patient has been free of any recurrences for 1.5 years since the last biopsy was taken at the time she came to our clinic seeking treatment.

## 1 INTRODUCTION

Oral eosinophilic ulcer is an inflammatory disease of the oral mucosa, featuring a painful ulceration with a tendency to heal spontaneously. This lesion is also known as traumatic ulcerative granuloma with stromal eosinophilia [1-3]. OEU occurs predominantly in individuals between their 5^th^ to 6^th^ decades, but there are also cases reported in young individuals. Both male and female are affected in equal frequency [1-4]. The cause and pathogenesis mechanism of this disease remain obscure.

Oral Eosinophilic Ulcer (OEU) usually affects the tongue but may involve any other site of the oral mucosa [1-5]. Clinically, it presents as a chronic ulcer with indurated borders that tend to persist for several weeks only to progress into remission usually after a surgical procedure, such as a biopsy. The biopsy procedure seems to accelerate the healing process, and is usually performed due to the resemblance of this lesion to a carcinomatous ulcer [1-4].

More often than not, patients exhibit a single episode with a single ulcerative lesion. Only a few reports of patients with recurrent episodes of OEU have appeared in the literature. This article highlights such a case in which a young woman was experiencing relapsing of chronic ulcerations on the oral mucosa.

## 2 CASE REPORT

**Fig. (1):**
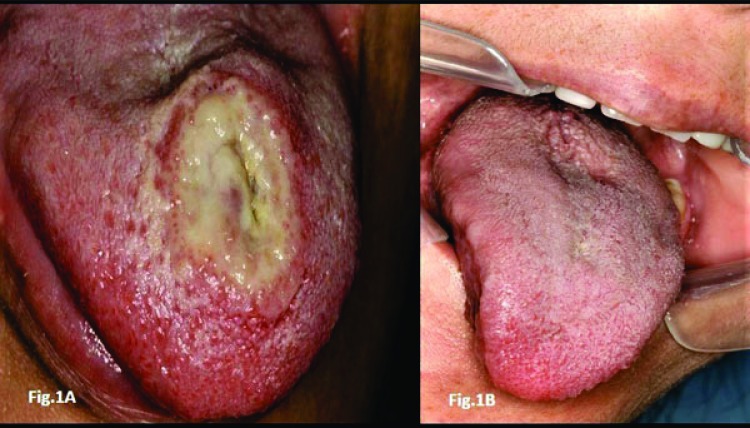
Initial presentation and resolution after biopsy. A typical lesion that supports a diagnosis of oral eosinophilic ulcers, *i.e.*, a large and rounded ulceration on the tongue’s dorsal surface. The ulceration also shows an elevated border and a yellowish exudate; 1B, – the photographic shows a completed healing of the ulceration along with a full re-papillation of the tongue within six weeks after a biopsy was taken.

**Figs. (2):**
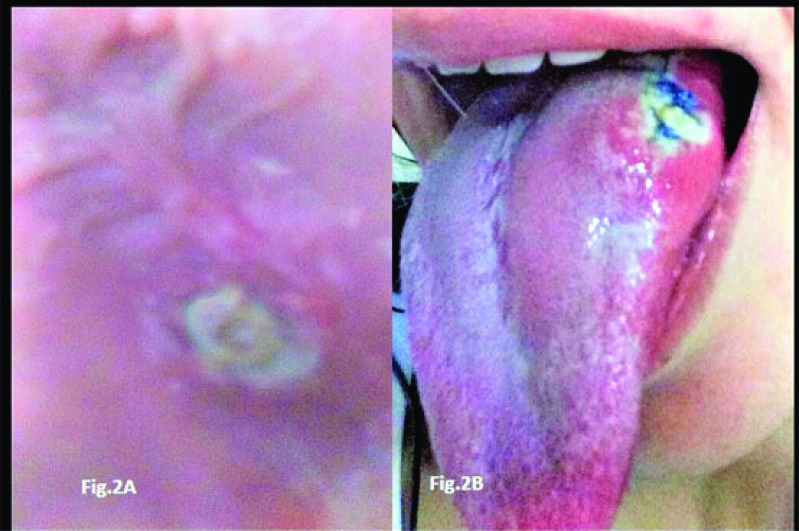
The recurrent aspects of the case both pictures were self-taken, one on hard palate and the other on the lateral border of the tongue, with the purpose of showing the recurrent nature of the present case.

A 31 years old white woman was referred to our oral medicine clinic for evaluation of a painful lesion on her tongue of three week’s duration (Figs. **1A**, **B**). The lesion was a fairly extensive ulceration with indurated and elevated borders. The current episode had started as an erythematous plaque with mild pain, which over the course of a few days, had progressed to a deep painful ulcer, and from that point onward had remained very painful precluding regular eating. Submandibular lymphadenopathy was present showing inflammatory features with mild symptomatology. The patient reported that she had been experiencing recurrent episodes of such ulcerations throughout the last 30 months. The lesions were self-healing with average duration of 20 days. The majority of the ulcers affected the tongue but she had one recurrence on the hard palate (Figs. **2A**, **B**). In one of the relapses, she went to a medical institution where she was submitted to a biopsy which revealed intense chronic inflammatory infiltrate, exuberant granulation tissue, and numerous eosinophil. In the last episode, she had experienced before that present one, she had also developed diarrhea, which prompted her to seek for an evaluation at a hospital. Under the suspicion of Crohn’s disease, the patient underwent a thorough laboratory examination along with endoscopy and colonoscopy. All exams revealed no abnormalities; she also tested negative for any type of immunosuppression, including negative test for HIV. Her medical history was that of a healthy person; she was neither a smoker nor a habitual consumer of alcoholic beverages.

## 3 DIFFERENTIAL DIAGNOSIS

A history of recurrent ulceration of the oral mucosa most likely leads to a diagnosis of Recurrent Aphthous Stomatitis (RAS). But this was ruled out based on both the clinical aspect (very large ulcerations) and, mainly, the location of the lesions, as they developed predominantly on areas of keratinized mucosa (hard palate and tongue’s dorsal surface) where RAS lesions very rarely occur. Additionally, the histopathological examination revealed the presence of eosinophils, which are not seen in RAS [6]. Another possibility was a recurrent intraoral herpes, but this would only be possible in immunosuppressive individuals, a condition that was not associated to the present case. Bullous autoimmune diseases bear no clinical similarity with the present case, and were consequently excluded. Crohn’s disease was possible but was later excluded as all examinations were negative for this disease during the patient’s admission to the hospital. Given the recurrent nature of the present case, malignancy of any type was, too, excluded. Therefore, the most probable diagnosis was OEU and a biopsy was taken. The subsequent histopathological examination of the specimen revealed features strongly compatible with OEU.

## 4 HISTOPATHOLOGICAL FINDINGS

**Figs. (3):**
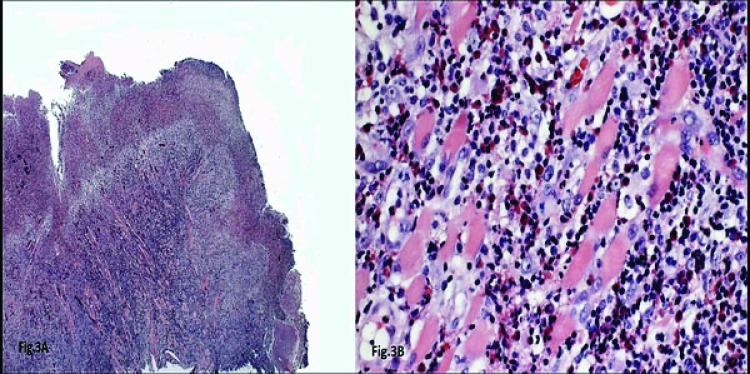
The histopathological characteristic s of the EU usually shows an ulcerated area covered with fibrinoid material (H&E, original magnification 40X).

Microscopic observation revealed a specimen of oral mucosa partially coated by a stratified squamous epithelium with an extensive ulcerated area covered by fibrin and neutrophils. The lamina propria was constituted by a dense connective tissue with an intense chronic inflammatory infiltrate constituted by lymphocytes and plasma cells that spread diffusely deep into muscle bundles. Eosinophils were frequently seen amidst the inflammatory infiltrate. This chronic inflammatory process with ulceration and eosinophilia is fully compatible with a clinical diagnosis of OEU (Figs. **3A**, **B**).

## 5 MANAGEMENT

Following the last biopsy, taken in our clinic a year and one-half ago, the patient has since then remained free of recurrences without the presence of any oral ulceration (Fig. **1B**). She has been periodically monitored and instructed to come our clinic at any time she noticed a recurrence.

## 6 DISCUSSION

A typical manifestation of OEU is a single ulceration occurring as a single episode. A well-known but poorly understood phenomenon in OEU is that the lesion usually heals following a biopsy procedure. A recurrent case of OEU is a very unusual manifestation although there have been some few reports of recurrent cases [6-8]. In this respect, there is a case report [8] in which the patient had as many as 30 episodes of recurrences and showed no benefit after multiple biopsy interventions.

One major controversial aspect about OEU relates to its etiopathogenesis. Trauma has been discussed in the literature as a major factor in OEU etiopathogenesis, but up to the present there has been no sound evidence supporting this correlation. In the present case, the patient had been experiencing many episodes of recurrences, with lesions appearing in different areas of the oral mucosa. This clinical behaviour would make it very unlikely that trauma had played any role in the development of the lesions. In fact, the role of trauma in OEU has not been clearly acknowledged in most of the studies [2, 4, 8].

Apart from the use of biopsy for a proper diagnosis of OEU, which is in itself the best treatment for the disease as it accelerates the healing process, other treatments such as topical or systematic corticosteroid have not shown conclusive evidence of their efficacy, since these medications are usually prescribed after biopsies or surgical excision. In the present case, although the patient was greatly benefitted by the last biopsy performed at our clinic, insofar as she was freed of recurrences, this does not mean that this procedure was in fact a cure for her disease, as she had been undergone to biopsies previously but without relief of recurrences.

On histopathological grounds, there is no specific feature that could point to OEU as a disease of neoplastic nature. Some authors [9, 10] have speculated on the possibility of OEU being the oral counterpart of the primary cutaneous lymproliferative disorder based on the positivity for CD30 protein (a cell-surface marker observed in many types of lymphoma). This hypothesis has not been confirmed (rather refuted) in one large study [1], in which a positivity for CD30 was seen in only 55% of 19 OEU cases analysed.

In this present case, as well as in other cases reported in the literature, patients usually show no signs of systemic abnormalities that could act as predisposing or trigger factors. The occurrence of the diarrhea in concomitance with one episode of oral ulceration reported in the current case was more likely a fortuitous combination rather than any cause/effect relationship. The cause of OEU remains utterly elusive –the affected patients develop this disease by no reason at all, and they may get scared, eventually. Fortunately, the disease is very uncommon, and, as the diagnosis is reached (a biopsy is mandatory for a conclusive diagnosis), a patient can be reassured that the lesion heals by itself after the biopsy and rarely recurs.

## CONCLUSION

Knowledge of this disease is overwhelming necessary since its lesion can mimic an oral squamous carcinoma occurring as an indurated ulceration coupled with its frequent occurrence on the tongue and targeting predominantly individuals at their 50s and 60s. The biopsy is not only the best approach for a proper diagnosis but also for its therapeutic management. Both the cause of this lesion as well as the reason it often disappears after a biopsy intervention remain an enigma.
